# Effect of Strabismus and Amblyopia on Postural Stability

**DOI:** 10.31486/toj.22.0104

**Published:** 2023

**Authors:** Ali Nouraeinejad

**Affiliations:** Department of Clinical Ophthalmology, University College London, London, United Kingdom

## TO THE EDITOR

Postural control is an individual's ability to achieve and maintain balance during a chosen posture or engaged activity.^1,2^ The 2 crucial functional aims of postural control are postural orientation (the active alignment of the trunk and head with respect to gravity, support surfaces, visual surround, and internal references by interpreting and applying sensory information) and postural stability (also known as postural equilibrium, the harmonization of sensorimotor strategies to stabilize the center of body mass throughout both self-initiated and externally triggered conflicts of stability).^1^

Postural stability is critical for the achievement of developmental milestones.^1,3^ Static (sitting and standing) and dynamic (walking and running) equilibrium, which are 2 subdivisions of postural stability, are essential for the normal development of gross and fine motor controls.^1,3^ Anomalous balance affects coordination of gross and fine motor controls in school-age children and leads to reduced academic performance and delayed social growth.^1,3^ Abnormal balance also has an impact on a child's general health, self-esteem, and safety.^1,3^

Postural stability depends on the central integration of 3 crucial sensory components: the visual, vestibular, and somatosensory (proprioceptive, cutaneous, and joint) systems and their rapid inputs for information processing.^1-4^

Apart from the sensory organization mechanism in which the central nervous system integrates the visual, vestibular, and somatosensory inputs, a motor tuning process, which necessitates the synchronization of musculoskeletal reflexes, and an internal representation of positions of body parts in space are required for successful postural control.^1,2^ These mechanisms work thoroughly to maintain a specific static state (eg, upright stance) or dynamic circumstance (eg, walking), as well as compensate for body perturbations to prevent falls.^1,2^

Although other sensory systems are engaged in maintaining the stability of the body in space, the role of vision is vital in postural control, especially in the upright position.^1^ The predominant role of vision in postural control has been confirmed by the findings of significant increases in postural instability in eyes-closed situations compared to eyes-open situations.^3,4^

Normal vision is critical in developing and maintaining balance control.^1,3^ When normal binocular vision is disrupted in childhood, especially by strabismus and/or amblyopia, balance is also affected.^1,3^ Reduced postural stability has been found in patients with strabismus and/or amblyopia in both static and dynamic (gait parameters) situations.^3,5^ In a cross-sectional cohort study, balance scores were profoundly reduced in participants aged 4 to 21 years in the amblyopia group (unilateral amblyopia including strabismic amblyopia or mixed mechanism) and the strabismus without amblyopia group (normal visual acuity in both eyes) compared to visually normal controls, but no statistical difference was found between the 2 patient groups.^3^ Postural instability can even occur in patients with intermittent strabismus with good stereopsis.^3^

These findings have important clinical implications for patients with strabismus and/or amblyopia who are already at a high risk of abnormal visual sensory and motor functions. This clinical point is even more important for children as they rely more intensely on the visual system to control their posture than adults do.^1-3^ However, because the issue of postural stability is currently overlooked in patients with strabismus and/or amblyopia during regular eye examinations, these patients are left unchecked for potential harm in their daily lives.

Therefore, the author urges all eye care professionals to consider clinical tests for balance performance in patients with strabismus and/or amblyopia or to refer their patients to relevant specialists for this testing. This recommendation highlights the crucial role of eye screening in children, as active therapeutic interventions can be efficiently initiated on time in an appropriate stage and help ensure that patients are referred early in life for proper management.
